# Not All Quiet on the Atherosclerosis Front

**DOI:** 10.3390/ijms24087527

**Published:** 2023-04-19

**Authors:** Katrin Schäfer

**Affiliations:** Department of Cardiology, Cardiology I, University Medical Center Mainz, 55131 Mainz, Germany; katrin.schaefer@unimedizin-mainz.de; Tel.: +49-6131-17-4221

In recent decades, research has identified the key cellular processes that take place during atherosclerotic plaque development and progression, including endothelial dysfunction, inflammation and lipoprotein oxidation, which result in macrophage and mural cell activation, death and necrotic core formation [1]. Nevertheless, many of the cellular and molecular mechanisms underlying these processes are yet to be revealed. **Endothelial dysfunction** is the earliest sign of atherosclerotic vascular alterations and is already present when the disease is clinically silent and reversible, highlighting the importance of its early detection [2,3]. The concept that cellular activation results in the shedding of cell components, including receptors and adhesion molecules or proteins stored in granules (which can be detected in the circulating blood and used as biomarkers of disease activity), also applies to a more complex form of cellular components: **extracellular vesicles** (EVs). Because EVs carry components of the cell from which they originate, the activation state of specific cell types can be assessed; however, methodologies to accurately quantify and characterize EVs are not available in all laboratories, and this approach is therefore not useful as a routine diagnostic tool. Moreover, and as Konstantinos Zifkos and colleagues point out [4], EVs are not only biomarkers, but may also transport their contents from one cell to another, thereby acting as initiators and propagators of cellular activation, including that observed in atherosclerosis. Importantly, EVs also hold great potential as therapeutic vectors that can deliver their cargo to sites of injury or to cell types of interest.

Inflammatory processes have long been known to play a key role in atherosclerosis initiation and progression [5]. Disease states associated with chronic, low-grade inflammation are associated with accelerated atherosclerosis progression. Patients with **systemic lupus erythematosus**, a chronic autoimmune disease that affects several organs, are also at increased risk of cardiovascular disease and mortality [6]. However, the experimental analysis of its pathomechanisms is hampered by the lack of specific animal models of the disease. Marczynski and co-workers employ lupus-prone MRL-*Fas*^lpr^ mice [7] to study pathological and functional changes in their vasculature, including vascular and perivascular inflammatory cell infiltrates in the aorta [8]. In addition to demonstrating the usefulness of this model for further research on SLE-associated vascular damage, the authors show that the resulting vascular dysfunction is ameliorated in MRL-*Fas*^lpr^ mice who lack **interleukin-6**. IL6 is a central cytokine produced by cells of the innate immune system as a result of inflammasome and IL1β activation [9]. As clinical trials using monoclonal antibodies that target IL1β have yielded promising results [10], the findings presented in this pre-clinical study support the possibility that targeting the inflammasome and its downstream effectors could also reduce vascular inflammation in specific patient populations.

One of the histological features of advanced atherosclerotic lesions is the presence of **calcification**. Vascular calcification is an actively regulated process that involves the osteo-genic transdifferentiation of smooth muscle cells, for example, in response to intraplaque hemorrhage [11,12]. Vascular calcification is not only a complication of atherosclerosis that is associated with plaque instability, but may also actively promote endothelial dysfunction, the earliest sign of vascular disease. In their work, Daria Shiskova and colleagues show that **calciprotein particles** (that is, colloidal nanoparticles formed via the binding of ionized calcium and phosphate to the mineral chaperone fetuin-A [13]) are internalized by endothelial cells, where they promote the expression of proinflammatory adhesion molecules and the recruitment of monocytes [14].

Although vascular occlusive disease is the most common form of atherosclerosis, up to 10 % of all individuals older than 65 years develop **aortic aneurysms** [15,16]. The increased activity of matrix metalloproteinases (MMPs) and the secretion of proteolytic enzymes from inflammatory cells are central components of its pathobiology. The current experimental and clinical evidence on MMPs and their potential as biomarkers in atherosclerosis are summarized by Olejarz, Lacheta and Kubiak-Tomaszewska [17]. Moreover, Jeremy Lagrange and co-workers show that mice who lacked **low-density lipoprotein-related protein-8**, also known as apolipoprotein E receptor-2, more frequently developed aortic aneurysm ruptures and exhibited increased mortality in response to angiotensin II. Although blood pressure and vascular inflammation did not differ, transplantation experiments suggested the involvement of bone marrow-derived cells, presumably macrophages, in the process [18]. In addition to describing a new mouse model of accelerated aortic aneurysm formation, their findings strengthen the link between inflammation and lipid receptor signaling in atherosclerosis complications.

Atherosclerotic plaque rupture, which can result in platelet activation, arterial thrombosis and obstruction of the coronary or carotid artery, is the pathological correlate underlying myocardial infarction or stroke, which are major complications of advanced atherosclerosis and the main causes of cardiovascular morbidity and death. Evidence that **platelet activation** is modified by **microbiota residing inside the gut** is provided by Christoph Reinhardt and his group, who show that platelets isolated from mice who lack gut microbiota from birth are less responsive in terms of adhesion or fibrinogen receptor expression [19]. Platelets are not in direct contact with the gut microbiome, at least in healthy states and in the presence of an intact endothelial cell barrier; however, microbiome factors are cleared into the liver via the portal vein, leading to the increased expression of pathogen-recognition receptors, such as Toll-like receptor 2, and the altered expression of coagulation proteins, such as von Willebrand factor [20]. The new data and previous work highlight the importance of the host microbiome as a disease modifier, but also as a therapeutic target, and the central role of the **gut–liver axis in atherosclerosis** [21,22].

Previous research has documented the contribution of specific cell types, such as endothelial cells, inflammatory cells or platelets and their products, in the pathophysiology of atherosclerosis. There is increasing evidence that dysfunction of the intrinsic biological pacemakers and molecular clock genes that coordinate cellular processes contributes to the development of vascular disease. Recent data on this topic and the role of **circadian rhythm control mechanisms** in the progression of atherosclerosis and thrombosis are presented and summarized by Andy Man, Huige Li and Ning Xia [23].

In addition to experimental studies on single disease candidates using mice who lack or overexpress the gene of interest, broader approaches are necessary to delineate pathways or signaling hubs and networks. To achieve this, it is important for researchers to undertake unbiased approaches to determine RNA and protein expression profiles, their temporal and spatial alterations and expression signatures at the single cell level. The results of (and the methodology underlying) **proteomic studies** on atherosclerosis, particularly the identification of potential biomarkers via the proteomic analysis of blood or vascular wall material from patients with coronary or carotid artery atherosclerosis, are discussed by Ekaterina Mikhailovna Stakhneva, Evgeniia Vitalievna Striukova and Yulia Igorevna Ragino [24]. Further, updates on the **key proteins and regulatory pathways in atherosclerotic plaque progression**, as well as on novel cellular pathways and effectors, including epigenetic regulation, biophysical factors and microorganisms that affect atherogenesis, are given in a mini-review by Michal Kowara and Agnieszka Cudnoch-Jedrzejewska [25]. The ways in which knowledge derived from experimental and clinical studies can improve patient care and diagnosis are exemplified in a review article by Tommaso Gori that focuses on nitric oxide (NO), a central mediator in vascular function control [26], and **current approaches to exogenously delivering or targeting NO signaling pathways** for the treatment and prevention of atherosclerosis [27].

In summary, the original research and focused review articles collected in this Special Issue highlight novel mediators, pathways and control mechanisms in the pathobiology of atherosclerosis, and also underline new models and methodologies that can be used to address specific research questions in this area.
